# The effects of leukoreduction on canine blood unit weight and processing time

**DOI:** 10.1111/vec.13225

**Published:** 2022-06-17

**Authors:** Richard D. Trinder, Eva Lo, Karen R. Humm

**Affiliations:** ^1^ Department of Clinical Science and Services, The Royal Veterinary College University of London North Mymms Hertfordshire UK; ^2^ Attimore Veterinary Hospital Welwyn Garden City Hertfordshire UK

**Keywords:** blood banking, packed red blood cells, transfusion reaction

## Abstract

**Background:**

Despite a lack of strong evidence of benefit, leukoreduction is employed to decrease the risk of leukocyte‐induced transfusion reactions. However, the impact of leukoreduction on blood bank costs and inventory management is not well understood. The purpose of this study was to determine whether leukoreduction of whole blood increases total processing time and weight loss from packed red blood cells (PRBCs) and plasma relative to bags created from nonleukoreduced whole blood.

**Key findings:**

A total of 68 canine whole blood collections were divided equally into leukoreduced and nonleukoreduced groups (N = 34 in each). There was no significant difference between groups in mean PRBC or plasma unit weights or processing times. Leukoreduced PRBC bags lost a significantly greater proportion of weight during processing than did nonleukoreduced PRBC bags (*P* < 0.01), which is attributed to red and white blood cells lost in the filtration process.

**Significance:**

Leukoreduction did not lead to a significant increase in processing times or smaller PRBCs or plasma bags compared to nonleukoreduced bags. The blood remaining in the leukoreduction filter following filtration is primarily composed of red blood cells, with minimal plasma retained.

**Abbreviations**
LR: leukoreduced
Non‐LR: nonleukoreduced
pRBC: packed red blood cells
WB: whole blood

## 1 INTRODUCTION

Blood product transfusions are widely used in veterinary patients^1^; however, their use is not without risk; transfusion reactions are reported in 3.3%–28% of canine recipients.^2^ The most common of these reactions are febrile nonhemolytic transfusion reactions (FNHTRs), accounting for 52% of all canine transfusion reactions in 1 retrospective study (which found an overall acute transfusion reaction frequency of 22%).^3^

Leukoreduction (LR), the process of removing leukocytes, with or without platelets, from whole blood (WB) has been implemented in human blood banks in over 20 countries^4^ as a means of reducing inflammation and potentially adverse reactions in transfusion recipients. A survey of private and teaching veterinary hospitals, however, reported that only 4% (2 of 53) used the filters routinely, although commercial blood banks were not included in this study.^1^ When used prior to storage, LR can effectively remove leukocytes from donated blood products and reduce the accumulation of cytokines while in storage.^5^, ^6^, ^7^, ^8^ The clinical benefits of LR and its ability to reduce the incidence of adverse transfusion reactions remain contentious in human medicine.^9^ Similarly, in the limited studies available in veterinary patients, LR has demonstrated limited or no detectable clinical benefit.^8^, ^10^, ^11^, ^12^

Although the possible benefits of LR are often discussed, the potential impact on blood donor inventory management and costs are not well described in the literature. A previous experimental study of 6 canine blood donations demonstrated that the process of LR resulted in a loss of approximately 52 mL from the total volume of a standard canine whole blood unit.^13^ However, it is unclear whether this substantial volume reduction is the result of the loss of cellular components or plasma. Ultimately, this reduction in blood products could result in the requirement for additional units in some patients, potentially precluding some from treatment due to financial limitations. The act of filtration is known to prolong processing times in experimental settings with increases of approximately 7–15 minutes (depending on blood temperature at the time of filtration).^13^ In addition, some licensed leukoreduction filters recommend the chilling and holding of products for 4 hours prior to processing, although there is marked variation between leukoreduction filters in terms of filtration rates, priming volumes, and filtration temperature.^14^ Whether this preparation, processing, and ultimately increased cost is evident in a clinical setting using a modern leukoreduction filter is currently not described in the literature.

The purpose of this study was to determine if proportionately greater weight (and therefore volume) is lost from packed red blood cells (PRBCs) and plasma units that have undergone LR filtration compared to units that have not and to estimate losses of cells and plasma to the LR filter. A secondary aim was to determine whether, in a veterinary hospital blood bank setting, the processing time of LR products differs from nonleukoreduced (Non‐LR) products. We hypothesized that canine blood products that have undergone LR would have a greater loss in volume and would take longer to process by veterinary staff when compared to non‐LR products.

## 2 METHODS

**FIGURE 1:**
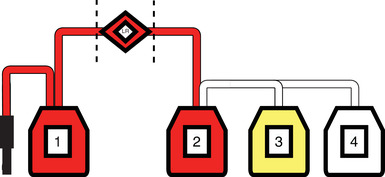
Diagram of the quad‐bag system. 1) Whole blood collection bag (WB‐1) 2) Whole blood bag following passage through or without leukoreduction (LR) filter (WB‐2). Dashed lines represent the points where the leukoreduction filter is aseptically ^removed^ and the lines aseptically rejoined in the nonleukoreduced group. Bag 2 is centrifuged and retains the packed red cell portion of whole blood. 3) Plasma is expressed from bag 2 into this bag following centrifugation (plasma). 4) Storage preservation is added to the packed red blood cells in bag 2 for final storage (PRBC)

All canine blood donations at Queen Mother Hospital for Animals, The Royal Veterinary College's University Teaching Hospital between October 2017 and March 2018 were enrolled in the study. Ethical approval was granted for this study by the university's Ethics and Welfare Committee (URN M2015 0054). Canine blood donors were above 25 kg and were deemed healthy through owner history and physical examination by a veterinarian. The hemoglobin of the donor was measured^a^ and recorded prior to blood collection. Blood donation was performed in a standard fashion^2^ into a quad bag system^b^ (Figure 1). Whole blood units were then randomized to either LR or non‐LR using a spreadsheet application.^c^ Units allocated to LR were placed in a refrigerator at 4°C for at least 1 hour prior to processing. The whole blood units (Bag 1) were then hung from a drip stand where filtration through the in‐line LR filter took place under gravity. Following the completion of the filtration, the whole blood (Bag 2) was centrifuged^d^ at 5000 relative centrifugal force at 4°C for 15 minutes. Once centrifuged, external pressure by a plasma extractor allowed the transfer of plasma to bag 3, which was then sealed and separated. Finally, a storage preservative solution^e^ was added to bag 2 (now containing PRBC) from bag 4. Units allocated to the Non‐LR group had the LR filter aseptically removed from the quad bag system using a heat sealer^f^ and sterile tubing welder^g^ that concurrently joined the two adjacent portions of tubing. The units underwent identical processing, although no filter was present in line between the primary collection bag (Bag 1) and Bag 2 of the Non‐LR group. The time taken for the process from hanging of the unit to the time the blood products were placed in storage was recorded.

All units were weighed after collection (WB‐1), once they had passed into Bag 2 of the quad bag system either via a leukoreduction filter or not (WB‐2) and after the production of PRBC and plasma. All weights recorded included both the contents and the bag itself. To compensate for the variation in initial WB‐1 volume, the weight of the WB‐2, PRBC, and plasma units were also calculated as a percentage of the WB‐1 by dividing the weight of the WB‐2, PRBC, and plasma units by their respective WB‐1 unit weights. These percentages were calculated to estimate proportional losses at each stage of processing, with the corresponding non‐LR values acting as controls.

### 2.1 Statistical analysis

Statistical comparisons were performed using a software spreadsheet program.^c.^ Data were tested for normality using a Kolmogorov–Smirnov Test of Normality, with unpaired *t*‐tests used to compare processing time, bag weights, and weight percentages between groups. Significance was determined as a *P*‐value < 0.05.

## 3 RESULTS

A total of 68 blood collections were included in the study and were equally divided between the LR and non‐LR groups (N = 34), with 42 males (27 neutered and 15 entire) and 26 females (20 neutered and 6 entire) recorded. A total of 24 different large and giant breeds were recorded, with the most frequent breeds including Labrador retrievers (13), greyhounds (6), cross breeds (5), and golden retrievers (4). The enrolled canine blood donors had a mean age of 5 years (range 1.4–9.1 years) at the time of donation, with a mean weight of 35.3 kg (range 25–64 kg). The mean hemoglobin concentration was 163 g/L (16.3 g/dL) (range 140–206 g/L [14–20.6 g/dL]). There was no significant difference in age, weight, or hemoglobin concentrations between the LR and non‐LR groups.

### 3.1 Product weights

The weight of the plasma unit was not recorded in 10 donations (5 in the LR group and 5 in the Non‐LR group). All other weights were collected for the 68 blood collections. The distribution of weights for all WB donations was found to be normal and ranged from 324 g to 604 g (428 g to 597 g for the LR group and 324 g to 604 g for the Non‐LR group, respectively). No significant difference in mean weight was demonstrated between the LR and Non‐LR groups for WB‐2, PRBC, and plasma products (Table 1).

**TABLE 1:** Mean (±standard deviation) bag weights during blood processing of leukoreduced (LR) and nonleukoreduced (Non‐LR) blood bags

	LR Group	Non‐LR Group	
WB‐1 (grams)	563.41 ± 39.90 (n = 34)	546.85 ± 70.15 (n = 34)	*P* = 0.24
WB‐2 (grams)	527.47 ± 37.97 (n = 34)	548.62 ± 71.59 (n = 34)	*P* = 0.14
PRBC (grams)	349.88 ± 26.17 (n = 34)	363.29 ± 42.39 (n = 34)	*P *= 0.12
Plasma (grams)	312.31 ± 22.32 (n = 29)	307.90 ± 41.26 (n = 29)	*P *= 0.61

WB‐1: Bag of whole blood immediately following collection.

WB‐2: Bag of whole blood that has been transferred to another bag either through or without a leukoreduction filter.

PRBC: Pack Red Blood Cells plus red cell preservative SAG‐M (100 mL).

When the percentage of weight relative to the WB‐1 unit was calculated, the LR group demonstrated a significantly (*P *< 0.01) greater reduction in weight than the Non‐LR group at WB‐2 (Table 2). There was also a significantly (*P *< 0.01) lower percentage weight for the PRBC units. However, there was no significant difference in the plasma percentage weight of WB‐1 (*P* = 0.51) (Table 2).

**TABLE 2:** Comparison of weight points of leukoreduced (LR) and nonleukoreduced (non‐LR) as a percentage of WB‐1

	LR Group	Non‐LR	
Intervals	Mean ± SD	Mean ± SD	Significance
WB‐2	93.61% ± 1.15% (n = 34)	100.29% ± 0.89% (n = 34)	*P* < 0.01
PRBC	62.31%± 5.22% (n = 34)	66.88% ± 6.06% (n = 34)	*P* < 0.01
Plasma	54.30 ± 4.31% (n = 29)	55.07 ± 4.49% (n = 29)	*P* = 0.51

WB‐1: Bag of whole blood immediately following collection.

WB‐2: Bag of whole blood that has been transferred to another bag either through or without a leukoreduction filter.

PRBC: Pack Red Blood Cells plus red cell preservative SAG‐M (100 mL).

### 3.2 Time of processing

When assessing processing times from the hanging of the unit to the storage of products between the LR and non‐LR groups, there was no significant difference (*P =* 0.15), with mean times of 140.71 (SD ± 63.88) minutes and 119.43 ± 59.02 minutes, respectively.

## 4 DISCUSSION

The purpose of this study was to determine whether LR of whole blood increases total blood processing time and results in smaller PRBC and plasma units relative to products created from non‐LR whole blood. When comparing the difference between the LR and non‐LR groups, there were no significant differences detected between the weights of PRBC, WB‐2, and plasma bags. This may have been because of type 2 error, as the study was likely underpowered to detect a difference.

A substantial variance was seen in the initial WB‐1 weight, which can be attributed to the study's reliance on nonsedated community voluntary donors, which therefore resulted in a heterogeneous population of WB unit weights. However, calculating weight points as a percentage of WB‐1 allowed for normalization of values, and therefore recognition of the effect of leukoreduction on PRBC unit weight can be seen (Table 2), with LR PRBC units being a smaller percentage weight of the initial WB‐1 unit. The sum of plasma and PRBC units was greater than the weight of WB units due to the addition of anticoagulant‐preservation solution (100 mL) to PRBC.

No significant difference was seen in the percentage weight of the plasma units relative to the WB‐1 units between the 2 groups. Although not previously recognized in the literature, the results are expected given that plasma is a mostly acellular product that is specifically processed to remove cellular material. However, there was a significantly (*P* < 0.01) greater percentage of weight loss when comparing the PRBC of each group. This shows therefore that the previously reported reductions in volumes of LR WB are predominantly due to a decrease in red cell volume.^13^

The assessment of processing time in this study demonstrated that no additional time was taken to produce LR units when compared to non‐LR units. Previous experimental evaluation of filtration time demonstrated that the act of filtration takes between approximately 7 and 15 minutes depending on the processing temperature.^13^ This current study demonstrated that in the context of this veterinary blood bank, the additional filtration time does not result in a significant difference in the processing of LR PRBC or plasma units. It is evident from the total processing time and substantial variance seen within both groups that in this clinical setting, many other factors other than filtration time influence processing time.

The present study had a number of limitations. By failing to sample packed cell volumes of the bags at each weight point, we were unable to truly quantify the loss of red blood cells at the various stages of processing. Due to a relatively low sample size, the study was likely underpowered to detect a difference in weights between LR and non‐LR PRBC units.

The present study did demonstrate that the LR process does not result in a greater loss of PRBC weight during processing or alter the total processing time in this clinical setting compared to non‐LR units. The study did, however, demonstrate that the majority of the weight lost during the LR process is predominantly due to a decrease in red cell volume. It is unclear whether the small change in PRBC red cell volume with LR filtration ultimately results in a requirement for additional units in patients, but given the low percentage decrease in weight, it is thought that in the majority of patients, this is unlikely. Although a statistically significant difference in percentage weight of PRBC relative to WB‐1 was demonstrated, given the wide range in PRBC unit weight overall, this difference does not appear clinically significant. Larger studies assessing the effect of LR blood products on canine blood product recipients in terms of transfusion reaction rate and outcome will help determine whether the cost:benefit of LR is worthwhile.

## CONFLICT OF INTEREST

The authors declare no conflicts of interest.

## ENDNOTES

a Hemocue Hb 201+, Hemocue, Ängelholm, Sweden.

b Composelect Blood Bag (PQ31555), Fresenius Kabi, Bad Homberg, Germany.

c Microsoft Excel 16.36, Microsoft Corporation, Redmond, WA.

d Sorvall RC3BP+, Thermo Fisher Scientific Centrifuge, Waltham, MA.

e SAG‐M, Fresenius Kabi, Bad Homberg, Germany.

f Composeal Universal, Fresenius Kabi, Bad Homberg, Germany.

g TSD‐II, Terumo, Tokyo, Japan.
